# Anti-C1q Antibodies as a Follow-Up Marker in SLE Patients

**DOI:** 10.1371/journal.pone.0123572

**Published:** 2015-04-16

**Authors:** Merete Bock, Ingmar Heijnen, Marten Trendelenburg

**Affiliations:** 1 Division of Internal Medicine, University Hospital Basel, Basel, Switzerland; 2 Division of Medical Immunology, Department of Laboratory Medicine, University Hospital Basel, Basel, Switzerland; Nippon Medical School Graduate School of Medicine, JAPAN

## Abstract

In cross-sectional studies autoantibodies against complement C1q (anti-C1q) were found to be highly associated with active lupus nephritis. The aim of this retrospective study was to determine the value of anti-C1q as follow-up marker of disease activity and renal involvement in patients with systemic lupus erythematosus (SLE). Fifty-two patients with SLE and a minimum of three anti-C1q measurements during follow-up were analyzed. Anti-C1q levels correlated with global disease activity scores. In subgroup analyses, patients without renal involvement did not show a significant correlation between anti-C1q levels and disease activity. In contrast, in patients with renal involvement, anti-C1q levels correlated well with global disease activity. In addition, a positive correlation with the urine protein-to-creatinine ratio and anti-dsDNA antibody levels as well as a negative correlation with complement levels was observed. Anti-C1q antibodies were found to strongly correlate with parameters of SLE disease activity during follow-up, in particular with regard to renal involvement.

## Introduction

Systemic lupus erythematosus (SLE) is the archetype of a systemic autoimmune disease taking a relapsing and remitting course. Immune dysregulation leads to the production of autoantibodies, immune complexes, complement activation and tissue inflammation, which together cause a clinical syndrome with multiorgan involvement and unpredictable courses [1]. Lupus nephritis, that occurs in about 50% of all SLE patients [2], is a common and severe complication, and considered to be a major cause of morbidity and mortality in SLE patients [3]. The large number of different autoantibodies observed in SLE mostly target nuclear as well as cell surface antigens, but also serum molecules such as complement components. Among these complement C1q is the most prominent target [4]. Complement C1q is the starter molecule of the classical pathway of complement activation and plays an important role in the clearance of immune complexes and apoptotic cell debris [5,6]. Interestingly, hereditary homozygous deficiency of C1q has been described to be the strongest risk factor for developing SLE [7–9]. Whereas most SLE patients do not suffer from hereditary C1q deficiency they often show very low levels of C1q, in particular during disease flares. Low levels of C1q are typically associated with the occurrence of autoantibodies against C1q [10–12] that are found in about 20–50% of unselected SLE patients and in up to 100% of SLE patients with active proliferative lupus nephritis [13, 14]. This strong association has also been described in pediatric-onset SLE patients [15, 16]. As a consequence, anti-C1q antibodies not only have a high negative predictive value for the occurrence of severe lupus nephritis but seem to be necessary (but not sufficient in themselves) for the development of proliferative lupus nephritis. However, although it is likely that they alter the physiological role of C1q, e.g. the uptake of immune complexes and apoptotic debris [17], the pathogenic role of anti-C1q still needs to be elucidated. Independently anti-C1q might serve as a biomarker of active lupus nephritis. This view is based on a number of cross-sectional studies on anti-C1q in which the antibody was found to have a significant association with renal involvement and general disease activity [18–22]. However, studies investigating the value of anti-C1q during clinical follow-up are scarce. In a large study, Moroni and colleagues followed patients with lupus nephritis during a period of 6 years measuring anti-dsDNA antibodies, C3, C4 and anti-C1q as markers of renal disease activity [23]. Anti-C1q levels were found to better correlate with renal flares in patients with proliferative lupus nephritis than the other markers, but not all patients with renal flares had increased levels of anti-C1q. In another study Akhter et al. followed patients with SLE and changes in renal disease activity showing an association between anti-C1q and changes in urine protein concentrations and a renal activity score as well as a modified SLEDAI [24]. In addition, it was reported that levels of anti-C1q antibodies decreased after successful treatment of lupus nephritis [25]. However, these findings are in contrast to data found by Katsumata et al. describing that anti-C1q antibodies were associated with SLE global disease activity but not specifically with active lupus nephritis [26]. Taken together, the value of anti-C1q in SLE patients as follow-up marker is controversial and data on the correlation between anti-C1q levels and changes in disease activity within individual patients are lacking. Therefore, the aim of this study was to determine the value of anti-C1q as a marker of disease activity in the follow-up of SLE patients with a focus on individual courses of the disease.

## Patients and Methods

### 2.1 Patients

In this retrospective study data from SLE patients followed at the University Hospital Basel or at the University Children’s Hospital Basel (Switzerland) between 1995 and 2013 were analyzed. As inclusion criteria, only patients fulfilling at least four of the eleven revised criteria of the American College of Rheumatology (ACR) for the classification as SLE [27]. In addition, with the aim to analyse anti-C1q in the follow-up, only patients with a minimum of three anti-C1q follow-up measurements were included. Because these measurements were part of the clinical care and therefore according to the physician’s judgment as well as to disease activity, intervals between follow-up time points were variable (between one month and 5,5 years). Patients were excluded from this study if clinical data at the time of anti-C1q measurement were not available or did not allow the classification of SLE. In total, this lead to the exclusion of seven patients from the initial screening cohort of 59 patients from which at least three anti-C1q measurements were available: Five patients did not fulfill at least 4 criteria for the classification of SLE. Another two patients having had three follow-up measurements of anti-C1q were followed in an external hospital and could not appropriately be verified with regard to the correct diagnosis of SLE.

Anti-C1q levels were compared to a number of blood, urine and clinical parameters. When the laboratory results were not available at the precise date of anti-C1q-measurement, values from up to 14 days before or after the measurement were accepted. Disease activity was assessed for each patient at each anti-C1q measurement point using the European Consensus Lupus Activity Measurement (ECLAM) score [28] as well as the Systemic Lupus Erythematosus Disease Activity Index (SLEDAI) [29]. The clinical disease activity scores were established retrospectively. Missing laboratory values were counted as 0 points, which occurred in about 2,5% (SLEDAI) and 5% (ECLAM) of all items respectively, and mostly concerned prot/crea ratios and urinary sedimentation. The study was approved by the ethical committee of the University Basel, Switzerland (Ref. Nr. EK 314/12). Patients records were anonymized and de-identified prior to analysis.

### 2.2. Measurement of anti-C1q and other laboratory parameters

All serum levels of anti-C1q were determined with the same commercially available ELISA kit (Bühlmann Lab., Schönenbuch, Switzerland) according to the manufacturer’s instructions. Anti-dsDNA antibodies were initially measured by Farr assay (IBL, Hamburg, Germany) until the end of 2009 when this method was replaced by a fluorescence enzyme-immunoassay (FEIA, Thermo Fisher, Freiburg, Germany). Due to this change of method, anti-dsDNA measurements were separated into two groups (i.e. before and after change of method) for further analyses. Levels of C3 and C4 were quantified by standardized nephelometric assay (Siemens, Marburg, Germany). CH50 was measured by using a modification of the method described by Mayer [30]. Blood count, erythrocyte sedimentation rate (ESR), creatinine levels, C-reactive protein (CRP), urine analysis and urine sediment all were determined by the routine laboratory procedures.

### 2.3 Statistical analyses

Demographic and clinical characteristics are reported as median and range or *n* (%), as appropriate. A nonparametric test (Spearman Test, one-tailed) was used to analyse possible correlations between biomarkers as well as between biomarkers and disease activity. Tests were performed using Graph Pad Prism (GraphPad Prism version 4.00 for Windows, GraphPad Software, San Diego California USA). P values of less than 0.05 were considered to be statistically significant.

## Results

### 3.1 Patients’ characteristics

In total, 52 patients with SLE each having at least three anti-C1q follow-up measurements were analyzed. The patient characteristics are summarized in Table 1.

**Table 1:** Patient characteristics.

	All patients	with renal involvement	without renal involvement
Number of patients	52	31	21
Gender, n (%)			
female	42 (81)	24 (77)	18 (85)
male	10 (19)	7 (23)	3 (15)
Age in years, median (range)	43 (14–69)	44 (23–69)	44 (14–69)
ACR-criteria fulfilled, median (range)	5 (4–9)	6 (4–9)	5 (4–8)
Measurement points per patient, median (range)	6 (3–30)	8 (3–30)	4 (3–18)
Biopsy-proven renal involvement	26 (50)	26 (84)	0 (0)
Patients with ANA+ (at any time) (%)	48 (92)	29 (94)	19 (90)
Patients with dsDNA+ (at any time) (%)	37 (71)	22 (71)	15 (71)

On average, the patients fulfilled 5 (median, range 4–9) ACR criteria for the classification of SLE. Forty-eight patients (92%) were positive for ANA and thirty-seven (71%) for anti-dsDNA antibodies. In these patients, a total of 460 anti-C1q measurements were identified and used for further analyses corresponding to a median of 6 data points per patients (range 3 to 29). The median age of the patients was 42 (range 14–68 years); ten patients (19%) were male and 42 female (81%). Twenty-six patients (50%) had a history of renal involvement as confirmed by renal biopsy, in another 5 patients renal involvement was descripted in the absence of available biopsy data.

### 3.2 Anti-C1q and activity indices

**Fig 1:**
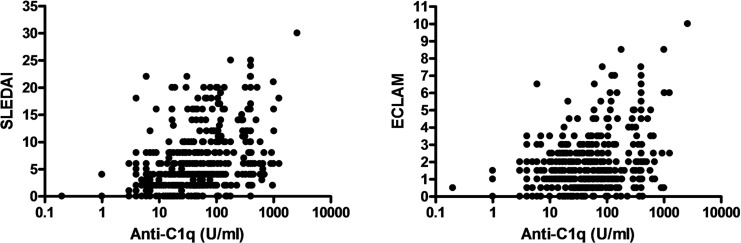
Correlation between Anti-C1q and disease activity indices for all patients and data points. SLEDAI: R = 0.43, p<0.0001 and ECLAM: R = 0.24, p<0.0001

With regard to disease activity, anti-C1q were found to positively correlate with disease activity as determined by ECLAM and SLEDAI scores (R = 0.24 and 0.43, respectively; p<0.0001 each). These data are demonstrated in Fig 1. For this correlation a significant albeit weak linear regression was found.

**Fig 2:**
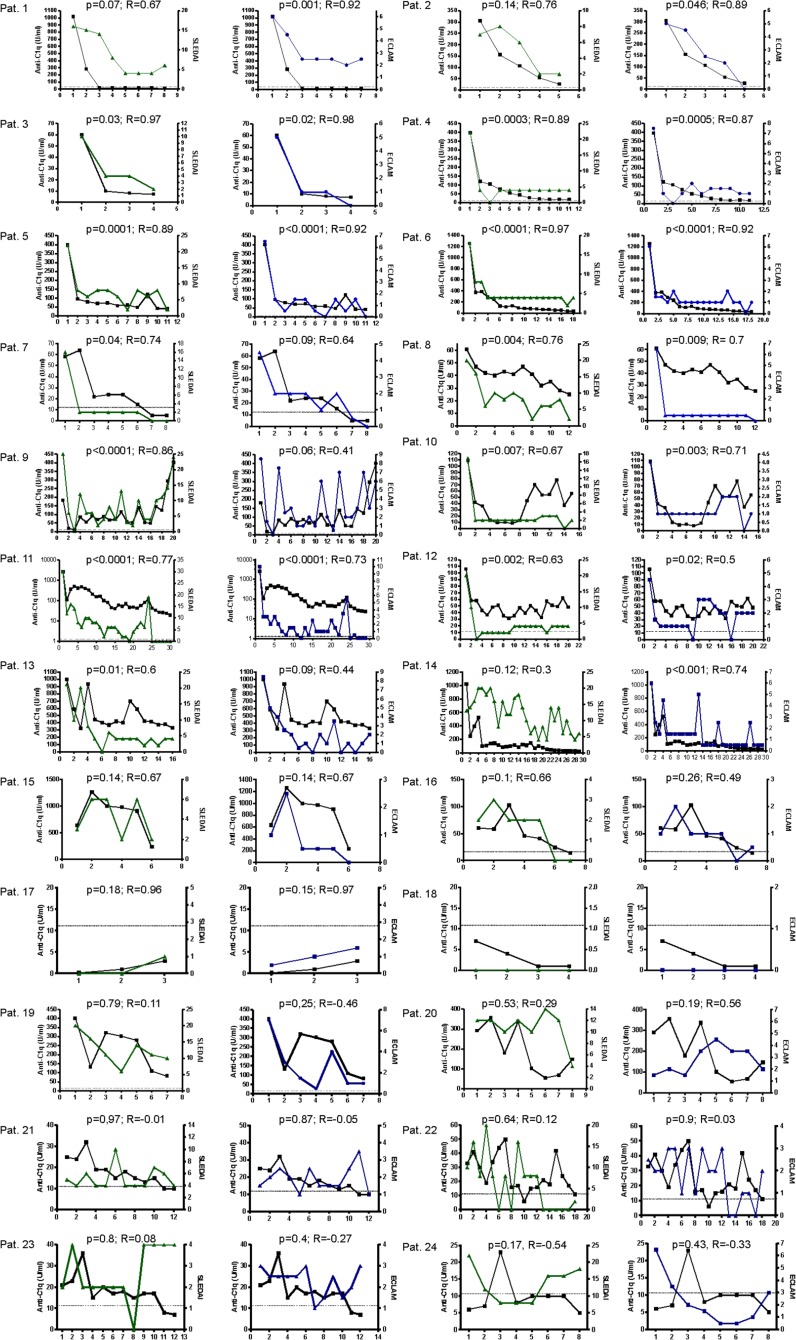
Exemplary courses of anti-C1q levels in relation to disease activity during follow up. (green: SLEDAI score, blue: ECLAM score, black: anti-C1q-level, x-axis: time points of measurement). Patients 1–8 showed an initial flare with high anti-C1q titers. After initiation of therapy disease activity as well as anti-C1q levels dropped reaching stable remission. Patients 9–16 showed flares during follow-up with either persistingly elevated levels of anti-C1q and/or a simultaneous increase of anti-C1q titers. Pat. 17 and 18 showed a stable low disease activity with concurrent low anti-C1q titers. Patients 19–24 showed a lack of correlation between disease activity and anti-C1q levels. Such a lack of correlation could be observed in patients with and without renal lupus.

Since anti-C1q levels varied considerably between patients with some having levels beyond the upper limit of detection whereas other patients remained low-level positive only, we evaluated individual anti-C1q levels. Characteristical follow-up curves are demonstrated in Fig 2.

Patients 1 through 8 presented at diagnosis with an initial flare and corresponding high disease activity associated with high anti-C1q levels (Fig 2a). After initiation of therapy disease activity as well as anti-C1q levels strongly decreased reaching stable values at remission with little disease activity and correspondingly low levels of anti-C1q. Patients 1 and 4 through 8 had biopsy-proven lupus nephritis at diagnosis, whereas patients 2 and 3 did not show apparent renal involvement.

Fig 2b summarizes patients with flares during follow-up with either persistently elevated levels of anti-C1q and/or a simultaneous increase of anti-C1q titers, as well as patients with stable low disease activity with concurrent low anti-C1q titers. Patients 9 through 16 had disease flares during follow-up or persistently high anti-C1q levels. Of these patients, patients 9 (with biopsy-proven proliferative lupus nephritis) and 10 (without apparent renal involvement) had several disease flares as indicated by increases of the activity indices and accompanying rises of anti-C1q levels followed by remission due to treatment adaptions. Patient 11 (with renal involvement) had persistently high levels of anti-C1q even during low disease activity. At measurement point 24 the patient was diagnosed to have a severe flare with secondary thrombotic thrombocytopenic purpura, mild renal failure and dilatative lupus-cardiomyopathy. At this time point a slight increase in anti-C1q levels beyond the high baseline levels could be detected. Patient 12–16 (with renal involvement) showed relatively stable disease activity and anti-C1q levels. Patients 17 (without renal involvement) and 18 (with renal involvement) were representative for patients with low disease activity associated with negative/very low anti-C1q levels.

However, not in all patients anti-C1q levels were found to clearly correlate with disease activity. As demonstrated in Fig 2c, patients 19 and 20 (without renal involvement) as well as patients 21–24 (with renal involvement) had a lack of correlation between disease activity and anti-C1q levels. Such a lack of correlation could be observed in some of our patients and, as demonstrated in the figure, was independent from a history of renal lupus.

**Fig 3:**
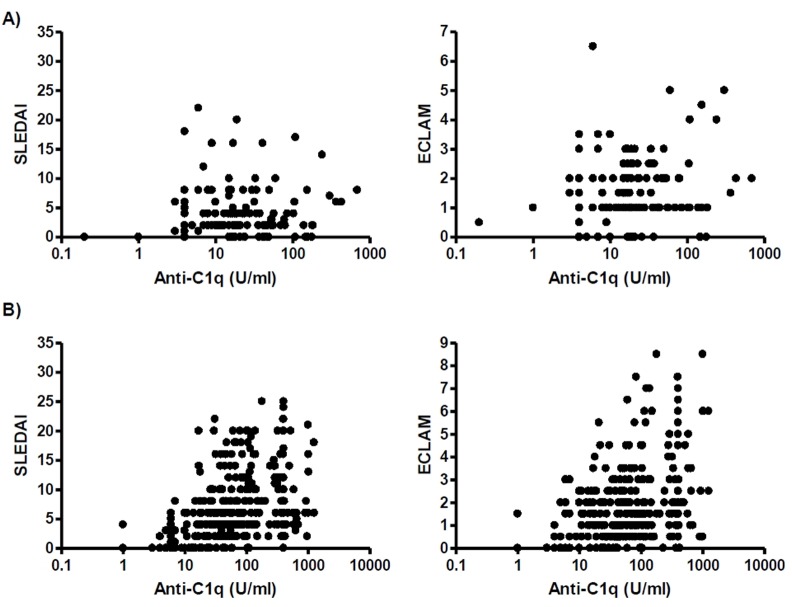
Correlation between anti-C1q and disease activity indices for (A) patients without renal involvement and (B) patients with renal involvement. Patients without renal involvement did not show a significant correlation between anti-C1q and activity indices (SLEDAI R = 0.05, p = 0.6; ECLAM R = 0.16, p = 0.07) whereas patients with renal involvement in the history showed a significant correlation between anti-C1q levels and activity indices (SLEDAI R = 0.47, p<0.0001; ECLAM R = 0.28, p<0.0001).

Since anti-C1q had been described to particularly well correlate with renal involvement [13–19], we subsequently separated the patients into two groups for further analysis: Group 1 comprising 31 patients with renal involvement (337 measurement points) and group 2 comprising 21 patients without apparent renal involvement ever (123 measurement points). For each group the correlation between anti-C1q levels and the activity indices were investigated separately (Fig 3).

Overall, in patients of group 2 (without renal involvement) no correlation between anti-C1q levels and disease activity indices was found (SLEDAI r = 0.05, p = 0.6; ECLAM r = 0.16, p = 0.07). In contrast patients of group 1 (with renal involvement in the history) showed a significant correlation between anti-C1q levels and disease activity indices (SLEDAI r = 0.47, p<0.0001; ECLAM r = 0.28, p<0.0001).

### 3.3 Anti-C1q and laboratory markers

**Fig 4:**
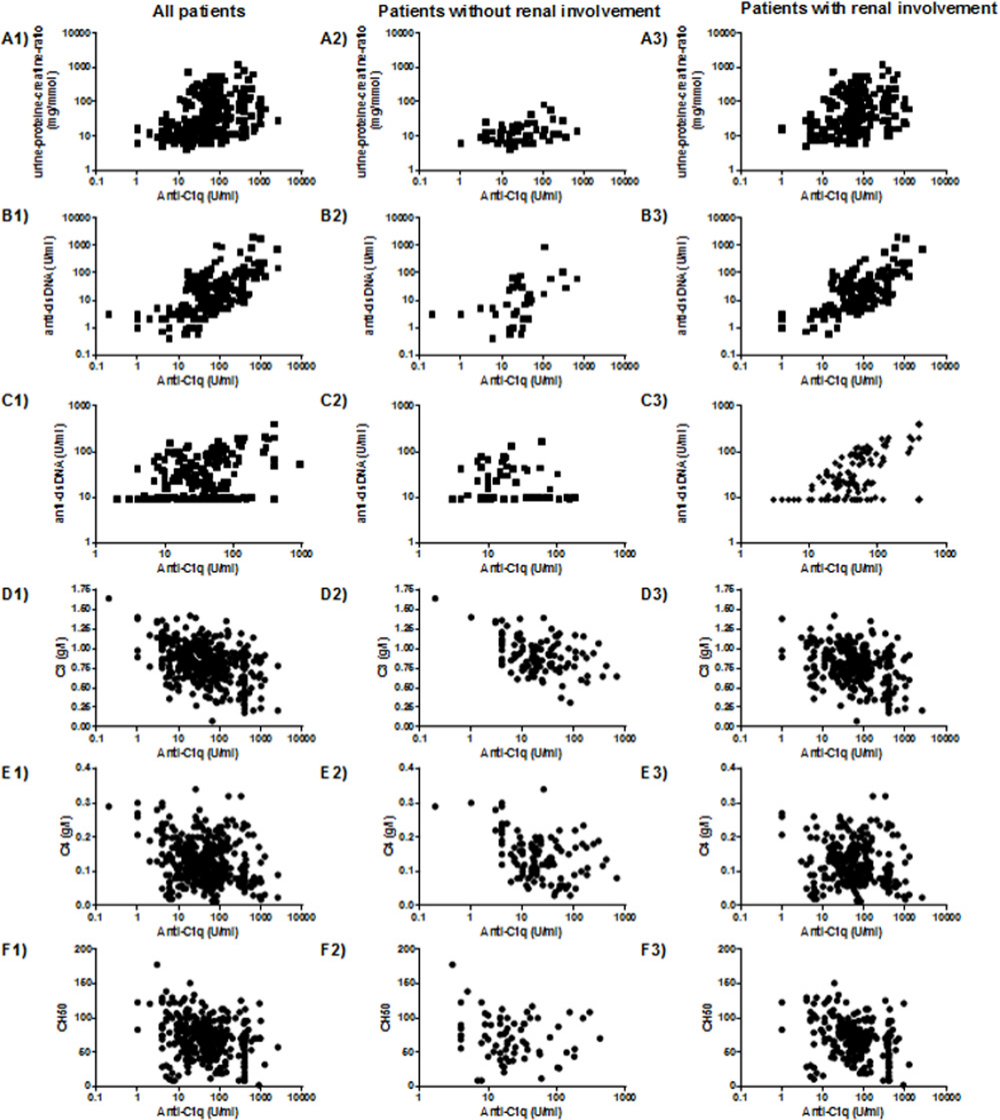
Correlation between anti-C1q and urine protein-to-creatinine ratio, anti-dsDNA antibodies and complement C3, C4, and CH50 for all patients, patients without renal involvement and patients with renal involvement. 1) = all patients, 2) = only patients without renal involvement, 3) = only patients with renal involvement (A) Correlation between anti-C1q and urine protein-to-creatinine ratio: (A1): R = 0.41, p<0.0001; (A2): R = 0.32, p = 0.01; p<0.0001 (A3): R = 0.28, p<0.0001; (B) Correlation between anti-C1q and anti-dsDNA measured with Farr assay: (B1): R = 0.59, p<0.0001; (B2): R = 0.4, p = 0.01; (B3): R = 0.6, p<0.0001; (C) Correlation between anti-C1q and anti-dsDNA measured with FEIA: (C1): R = 0.42, p<0.0001; (C2): R = 0.37, p = 0.002; (C3): R = 0.47, p<0.0001; (D) Correlation between anti-C1q and complement C3: (D1): R = -0.36, p<0.0001; (D2): R = -0.33, p = 0.0003; (D3): R = -0.3, p<0.0001; (E) Correlation between anti-C1q and C4: (E1): R = -0.24, p<0.0001; (E2): R = -0.35, p = 0.0001; (E3): R = 0.16; p = 0.006; (F) Correlation between anti-C1q and CH50: (F1): R = -0.26, p<0.0001; (F2): R = -0.42, p = 0.002; (F3): R = -0.3, p<0.0001.

The overall associations between anti-C1q levels and the laboratory parameters CH50, C3, C4, anti-dsDNA, and urine protein-to-creatinine ratio for all data points of the 52 included SLE patients are demonstrated in Fig 4.

Anti-C1q correlated positively with the urine protein-to-creatinine ratio (p<0.0001; R = 0.41) and anti-dsDNA antibody levels (Fig 4A1–4C1). With regard to correlations with anti-dsDNA antibody levels, anti-C1q correlated in both groups of anti-dsDNA assays, Farr assay as well as FEIA (R = 0.59 and R = 0.42, respectively; p<0.0001 each). The associations between anti-C1q and complement components C3 and C4 as well as CH50 are demonstrated in Fig 4D1–4F1. A negative correlation with all three complement parameters was found (C3: R = -0.36, p<0.0001; C4: R = -0.24, p<0.0001; CH50: R = -0.26, p<0.0001).

Interestingly, the correlations of anti-C1q with other laboratory markers could not only be observed in patients with confirmed (Fig 4A3–4F3) but also in those without confirmed renal involvement (Fig 4A2–4F2).

## Discussion

This retrospective follow-up study was performed to evaluate the diagnostic value of anti-C1q in the clinical follow-up of SLE patients with regard to disease activity in general and to renal involvement in particular. The focus of our analysis was on the time course of disease activity in relation to individual anti-C1q levels. We observed a good overall correlation between anti-C1q levels and disease activity indices (SLEDAI and ECLAM). In addition, this is the first study to demonstrate individual time courses of anti-C1q levels in relation to disease activity. Most SLE patients were found to have anti-C1q levels that well correlated with disease activity over time. Increasing or decreasing disease activity was accompanied by rising or falling anti-C1q levels in patients with and without renal involvement. In addition, a significant positive correlation between anti-C1q levels and the urine protein-to-creatinine ratio, anti-dsDNA antibodies as well as a negative correlation with complement C3, C4 and CH50 was observed. Interestingly, anti-C1q correlated not only with urine protein-to-creatinine ratio in patients with confirmed but also in those without confirmed renal involvement suggesting subclinical renal involvement. However, separating the patients with regard to renal involvement, only the patient group with renal involvement showed a significant overall correlation between anti-C1q levels and the activity indices suggesting that anti-C1q as a follow-up marker is best in SLE patients with renal involvement. This assumption is in line with the observation that the degree of correlation of anti-C1q levels is more pronounced with the SLEDAI than with the ECLAM score. This difference might be explained by an unequal weighting of renal involvement between both indices with renal involvement being stronger represented in the SLEDAI score than in the ECLAM score. Indeed, if items for renal activity were omitted in the SLEDAI score, the degree of correlation with anti-C1q levels strongly decreased (data not shown).

Previous studies suggested that the development or recurrence of nephritis might be associated with rising titers of anti-C1q during the six months preceding the flare suggesting that anti-C1q might serve as an early marker of renal flares [31,32]. In our study we could not observe such a rise in anti-C1q levels preceding increases in disease activity or urine protein-to-creatinine ratios. However, it is important to note that the intervals between follow-up time points varied considerably ranging from one month to several years in some patients. As a consequence, the question whether increasing disease activity is accompanied by rising anti-C1q levels preceding the flare by several weeks to a few months and thus predicting disease flares could not appropriately be addressed.

With regard to correlations of anti-C1q with other laboratory markers, it is noticeable that anti-C1q better correlated with anti-dsDNA levels determined by Farr assay than by FEIA. The difference between these two methods of measurement might be explained by the fact that the Farr assay primarily detects antibodies with high affinity for dsDNA whereas the FEIA method also detects low affinity antibodies. It is well established that high affinity anti-dsDNA antibodies are more specific since they better correlate with the occurrence of lupus nephritis [33]. This stronger association with renal involvement (which is typical for high anti-C1q levels too) as well as the fact that classical anti-C1q antibody assays are designed to detect high affinity antibodies only (by the use of a high ionic strength buffer) might well explain the strong correlation between anti-C1q and anti-dsDNA measured by Farr assay.

An important strength of our study is the large number of data points allowing the analysis of long follow-up periods of individual patients with SLE. The individual follow-up periods comprised at last three data points per patient in order to allow true follow-up analyses and reducing a bias towards a more cross-sectional character of the study. In addition, the single center character of our study allowed a uniform analysis of patient data due to a uniform documentation system. Furthermore, all anti-C1q levels were measured by the same medical laboratory employing the same assay during the whole study period, thus eliminating a potential interassay and interlaboratory variability. In this context, an important issue of anti-C1q measurements is its inadequate standardization and the lack of fixed cut-off levels discriminating positive from negative test results. In order to avoid this problem we performed correlation analyses independent from a specific cut-off for positivity.

On the other hand, our study bears some important limitations. The retrospective character of our analysis and the relatively small number of patients, in particular when comparing subgroups of patients, do not allow drawing definite conclusions. The study characteristics also did not sufficiently allow the calculation of the sensitivity and specificity of anti-C1q for the determination of a specific nature and severity of flares in comparison to other laboratory markers of disease. Furthermore, anti-C1q were measured according to the physician`s decision and therefore certainly were more often determined in patient with active disease than in patients without flare. Last, our analysis of the association of anti-C1q with renal disease activity mainly focused on the correlation of anti-C1q with prot/crea ratios. However, persisting stable proteinuria not necessarily is expression of active lupus nephritis but might also reflect chronic renal damage. Further studies will have to be performed using well-defined large cohorts of patients with close as well as long follow-ups in order to determine the diagnostic value of anti-C1q in SLE patients.

In conclusion, anti-C1q levels were found to well correlate with disease activity in SLE patients during follow-up. This correlation was strongest in patients with renal involvement and active lupus nephritis. Our data support the use of anti-C1q as follow-up marker in SLE patients, in particular but not exclusively in SLE patients with renal involvement.
